# Gut barrier and microbiota changes with glycine and branched‐chain amino acid supplementation in chronic haemodialysis patients

**DOI:** 10.1002/jcsm.12781

**Published:** 2021-09-18

**Authors:** Laurence Genton, Menno Pruijm, Daniel Teta, Isabelle Bassi, Patrice D. Cani, Nadia Gaïa, François R. Herrmann, Nicola Marangon, Julie Mareschal, Giulio G. Muccioli, Catherine Stoermann, Francesco Suriano, Arlene Wurzner‐Ghajarzadeh, Vladimir Lazarevic, Jacques Schrenzel

**Affiliations:** ^1^ Unit of Nutrition Geneva University Hospitals and University of Geneva Geneva Switzerland; ^2^ Service of Nephrology University Hospitals of Lausanne and University of Lausanne Lausanne Switzerland; ^3^ Service of Nephrology Cantonal Hospital of Sion Sion Switzerland; ^4^ Louvain Drug Research Institute, Metabolism and Nutrition Research Group, Walloon Excellence in Life Sciences and BIOtechnology (WELBIO) Université catholique de Louvain Brussels Belgium; ^5^ Genomic Research Lab and Service of Infectious Diseases Geneva University Hospitals and University of Geneva Geneva Switzerland; ^6^ Department of Rehabilitation and Geriatrics Geneva University Hospitals and University of Geneva Geneva Switzerland; ^7^ Service of Nephrology Geneva University Hospitals and Clinique of Champel Geneva Switzerland; ^8^ Louvain Drug Research Institute, Bioanalysis and Pharmacology of Bioactive Lipids Research Group Université catholique de Louvain Brussels Belgium; ^9^ Service of Nephrology Geneva University Hospitals and University of Geneva Geneva Switzerland

**Keywords:** Gut microbiota, Glycine, Branched‐chain amino acid malnutrition, Endocannabinoids, Appetite

## Abstract

**Background:**

We have previously shown that glycine increases fat‐free mass in chronic haemodialysis patients with features of malnutrition as compared with branched‐chain amino acids (BCAAs). This multicentre randomized double‐blind crossover study evaluates the impact of these amino acids on the gut barrier and microbiota.

**Methods:**

Haemodialysis patients were included if they had plasma albumin <38 g/L or weight loss >5% of dry body weight, and daily dietary intakes <30 kcal/kg and <1 g protein/kg. They consumed glycine or BCAA (7 g twice daily) for 4 months and underwent a 1 month washout period, before crossover of supplementations. Faecal microbiota (16S rRNA gene sequencing) and immunoglobulin A (IgA), serum levels of cytokines, surrogate markers of intestinal permeability, appetite mediators, and endocannabinoids were obtained at the start and end of each supplementation. Supplementations were compared by multiple mixed linear regression models, adjusted for age, sex, month of supplementation (0 and 4 in each period), and period (Period 1: first 4 months; Period 2: last 4 months). Microbiota comparisons were performed using principal coordinate analysis and permutational multivariate analysis of variance, Shannon diversity index estimate and analysis of composition of microbiomes analysis, and Wilcoxon tests.

**Results:**

We analysed 27 patients compliant to the supplementations. Multiple mixed linear regression models were significant only for interleukin‐6 (*P* = 0.002), glucagon‐like peptide 1 (*P* = 0.028), cholecystokinin (*P* = 0.021), and peptide YY (*P* = 0.002), but not for the other outcomes. The significant models did not show any impact of the type of supplementation (*P* < 0.05 in all models). Principal coordinate analysis and permutational multivariate analysis of variance (*P* = 0.0001) showed strong microbiota clustering by subject, but no effect of the amino acids. Bacterial alpha diversity and zero‐radius operational taxonomic unit richness remained stable, whatever the supplementation. *Lacticaseibacillus paracasei* (0.030; Q1–Q3 0.008–0.078 vs. 0.004; Q1–Q3 0.001–0.070) and *Bifidobacterium dentium* (0.0247; Q1–Q3 0.002–0.191 vs. 0.003; Q1–Q3 0.001–0.086) significantly decreased with the BCAA supplementation.

**Conclusions:**

The BCAA and glycine supplementations had no impact on the serum levels of cytokines, appetite mediators, intestinal permeability, endocannabinoids, or faecal IgA. Overall faecal microbiota composition and microbial diversity did not change with the glycine or BCAA supplementation but decreased the abundance of *L. paracasei* and *B*. *dentium*.

## Introduction

Muscle wasting occurs in 30–55% of chronic haemodialysis (HD) patients.^1^ It results from an increased protein degradation and a reduced protein synthesis and myogenesis. Reduced skeletal muscle mass in HD patients has been associated with impaired physical function, low quality of life, longer hospital stays, and increased mortality,^2^ emphasizing the importance of maintaining muscle mass in order to improve the clinical outcome. Muscle wasting can be limited by the treatment of co‐morbidities and by the optimization of calorie and amino acid intakes.^3^

Several animal studies have linked the muscle mass to the gut microbiota. For instance, germ‐free mice have skeletal muscle atrophy, but their muscle mass and function improve when transplanted with the faecal microbiota of pathogen‐free mice or when treated with bacteria‐derived metabolites such as short‐chain fatty acids.^4^ Similarly, muscle mass can increase in response to the enteral administration of *Escherichia coli* or *Lactobacillus reuteri* in mouse models of diseases associated with low muscle mass.^5^, ^6^ Finally, a translational study reported a lower weight gain in mice transplanted with the faeces of underweight children as compared with the faeces of healthy children.^7^ The authors identified a consortium of bacteria that distinguished the two mice groups. When the mice transplanted with the faeces of the underweight children received additionally the bacterial consortium, they increased their lean body mass. These studies highlight the importance of gut microbiota in the modulation of the muscle mass.

The mechanisms behind this modulation have not yet been unravelled, but several hypotheses, based on animal studies, have been reported.^8^ An alteration in the composition and function of the gut microbiota, sometimes referred to as dysbiosis, could lead to muscle wasting by metabolizing the ingested amino acids, thereby decreasing the amino acid bioavailability for the muscle, or by producing microbial metabolites involved in increased energy expenditure.^8^ It could also promote muscle wasting via its impact on the intestinal barrier function. The latter, when impaired, may be accompanied by increased translocation of bacteria or pathogen‐associated molecular patterns as lipopolysaccharides (LPS), which may stimulate the gut‐associated lymphoid tissue and lead to systemic inflammation. Indeed, diseases prone to muscle wasting are often associated with increased intestinal permeability and systemic inflammation.^9^ The gut microbiota could also influence body weight and muscle mass by affecting appetite mediators, mostly synthesized in the digestive tract.^10^ Among them, the neuropeptide Y and ghrelin are known as orexigenic, while the cholecystokinin (CCK), leptin, peptide YY (PYY), glucagon‐like peptide 1 (GLP‐1), and *N*‐oleoylethanolamine are anorexigenic. Moreover, it is still unknown whether an altered profile of the bioactive peptides produced and secreted by the muscle (i.e. myokines) can further affect the composition of the gut microbiota. Interestingly, the endocannabinoid system (i.e. the endocannabinoids and the associated *N*‐acylethanolamines) is known to crosstalk with the gut microbiota and to regulate several key functions including gut permeability and energy expenditure.

This study evaluates (i) whether the supplementation with glycine or branched‐chain amino acids (BCAAs) is associated with changes in gut barrier and microbiota and (ii) whether these changes differ between the supplementations. Primarily, this study was expected to link a BCAA‐induced increase of fat‐free mass with the gut microbiota, vs. glycine as placebo. However, glycine, and not BCAA, increased fat‐free mass in HD patients.^11^ Thus, we hypothesize that oral glycine increases fat‐free mass through changes of the gut microbiota, decreases gut permeability and systemic inflammation, and changes the balance of appetite mediators in favour of orexigenic hormones and increases the serum levels of endocannabinoids.

## Study population and methods

This multicentre randomized double‐blind crossover study evaluated the impact of BCAA and glycine on the gut microbiota as primary outcome and on systemic inflammation, gut permeability, and faecal IgA as secondary outcomes. The trial design was chosen in view of the large inter‐individual variability of the gut microbiota. In a parallel study, it would not have been possible to differentiate whether the changes in microbiota were related to the supplementations or to differences in baseline microbiota. It lasted from 1 August 2016 to 31 August 2019. The participating centres were the Geneva University Hospitals, the Clinic of Champel Geneva, the University Hospital of Lausanne, and the Cantonal Hospital of Sion, Switzerland. The local ethics committees accepted the protocol, which was registered under clinicaltrials.gov (NCT 02962089), and all participants signed an informed consent.

### Patient selection

All patients followed in one of the four previously mentioned haemodialysis centres and matching the inclusion criteria were asked for informed consent by the study investigators. Patients were included if they had been undergoing HD for ≥3 months, had not received systemic antibiotics in the previous month, and had features of malnutrition such as pre‐dialysis plasma albumin measured by bromocresol green <38 g/L without any known acute infection during the previous 2 weeks, and nutritional intakes <30 kcal/kg/day and <1 g protein/kg/day based on a 24 h dietary recall.

We excluded patients with the following conditions: known psychiatric or cognitive disorder; history of poor compliance with medication or HD treatment; life expectancy ≤1 year; single pool inadequate dialysis (Kt∕V < 1.2 on three consecutive occasions); known reasons for decreased plasma albumin levels such as liver failure or exsudative enteropathy; enteral or parenteral nutrition; drugs or oral nutritional supplements containing fibres since ≤1 month; drugs influencing body composition since ≤1 month, as systemic corticosteroids, anabolic drugs such as insulin or testosterone, postmenopausal hormone therapy, and injectable contraceptives; known disorders potentially leading to hypometabolism or hypermetabolism, untreated or treated since ≤1 month, as disorders of thyroid or adrenal glands; or pregnancy and breastfeeding.

The maximum time lap between the screening and the signature of the informed consent was set at 2 weeks, to allow the patient to have sufficient time to confirm his or her participation and to address all his or her questions. The maximum time lap between screening and baseline tests (and start of the study treatment) was 6 weeks. This time lap was necessary for the organization and the collection of all baseline tests.

### Glycine and branched‐chain amino acid supplementation

Both supplements were conditioned as granules in packs of 7 g (Pharmacy Bichsel, Interlaken, Switzerland) and were similar in appearance and taste. One glycine pack contained 7 g glycine, while one BCAA pack contained 3.62 g leucine, 1.45 g isoleucine, and 1.94 g valine.

The included patients were randomized to consume glycine or BCAA for 4 months, undergo a 1 month washout period, and then to take the other supplement for 4 months. Thus, the study lasted 9 months for each patient. The glycine–BCAA group was defined as the group that started the study with the glycine supplement and the BCAA–glycine group as the one that started with the BCAA supplement.

The patients were asked to take two packs daily, one 30 min before breakfast and another 30 min before lunch. They were compliant to the protocol if they had consumed >80% of the supplements over each 4 month period. This was checked monthly by the research assistants who counted the number of empty and full packs that the patient was bringing back.

### Study randomization

The manufacturer sent the packs of supplements to the pharmacy of the University Hospitals of Geneva, after having assigned one number to the BCAA packs and another number to the glycine packs, as well as the type of supplement assigned to each number. The statistician (F. R. H.), blinded to the type of supplement assigned to each number, generated two lists of randomization through the Stata software (Stata version 16.1, StataCorpLP, College Station, Texas, USA), with the method of randomly permuted blocks with random block sizes of 2 and 4. Sequence assignment was randomized separately for the hospitals of Geneva, Lausanne, and Sion to ensure an equal percentage of supplementation sequences in the three centres and communicated to the pharmacy, who assigned the supplementations to the participants. The clinic of Champel was added as study centre in October 2017, to accelerate the recruitment. We could therefore not perform a separate randomization of sequence assignment for this centre and decided to take the two last treatments attributed to Lausanne and the four last treatments attributed to Sion. All participants, caregivers, investigators, and the statistician were blinded to the type of supplement. Unblinding was performed only after the first set of analysis.

### Measured outcomes

For each included patient, we recorded sex, date of birth, medical history, and medications at baseline and during follow‐ups. The outcomes were measured at the start and at the end of each supplementation, thus four times for each patient. Of note, the information regarding baseline body composition, routine blood parameters and plasma amino acid profile, nutritional intakes, appetite, physical function and quality of life, and their changes with the supplementations is available in another paper.^11^

#### Blood analyses

All blood samples were taken in the fasted state. Serum samples were used to assess systemic pro‐inflammatory [interleukin‐6 (IL)‐6 and tumour necrosis factor (TNF)‐α] and anti‐inflammatory (IL‐10) cytokines, gut permeability [LPS and glucagon‐like peptide 2 (GLP‐2)], and appetite mediators (total ghrelin, active ghrelin, leptin, active GLP‐1, CCK, neuropeptide Y, and PYY). LPS and GLP‐2 were used as surrogate markers of gut permeability as it cannot be assessed through the urinary lactulose/mannitol ratio in HD patients who are potentially anuric. Low plasma LPS has been strongly correlated with decreased intestinal permeability assessed by urinary sugar concentration in several diseases.^12^ GLP‐2, a peptide synthesized by the enteroendocrine L cells, decreases gut permeability in rodents^13^ and reduces faecal weight and calorie excretion in humans with short bowel syndrome.^14^

Blood samples for measurements of cytokines and hormones were collected in EDTA tubes (BD Vacutainer^®^, BD, Eysins, Switzerland), placed on ice and taken immediately to the lab, centrifuged at 10 min at 2000 *g* to obtain the sera, which were stored in cryotubes at −80°C until analysis. Analyses of cytokines and hormones were performed by enzyme‐linked immunosorbent assay, according to the instructions of the manufacturer: IL‐6, IL‐10, TNF‐α, total ghrelin, leptin, total GLP‐1, and PYY by U‐PLEX metabolic group assays (MSD, Rockville, MD, USA); CCK by Antibodies‐online (Aachen, Germany); GLP‐2 and NPY by Merck (Darmstadt, Germany); and active ghrelin by Gentaur (Kampenhout, Belgium).

For the measurement of LPS, blood was collected in 5 mL SST tubes (BD Vacutainer, BD), also centrifuged at 10 min at 2000 *g*. The sera were transferred in Axygen cryotubes with sterile Axygen Maxymum Recovery tips (Fisher Scientific AG, Reinach, Switzerland) and stored at −80°C until analysis. LPS levels were measured by using a competitive inhibition enzyme immunoassay (Cloud‐Clone Corp., Houston, TX, USA). Samples were diluted 1/10 in dispersing reagent and heated 15 min at 70°C. Samples displaying haemolysis were excluded from the analysis according to the manufacturer's instructions. All determinations were performed in duplicate.

Following liquid–liquid extraction, endocannabinoid levels were determined by ultraperformance liquid chromatography–tandem mass spectrometry.^15^ Briefly, the samples were extracted using dichloromethane, methanol, and water (using a 4:2:1 ratio) in the presence of deuterated internal standards, dibutylhydroxytoluene and EDTA. Subsequently, the extracted lipids were purified by silica‐based solid‐phase extraction,^16^ and the resulting purified fraction was analysed using a Xevo TQ‐S mass spectrometer coupled to an Acquity UPLC class H. The endocannabinoids were separated using an Acquity UPLC BEH C18 (2.1 × 50 mm; 1.7 μm) column and a gradient between MeOH:H_2_O;acetic acid (75:24.9:0.1; v/v/v) and MeOH:acetic acid (99.9:0.1; v/v). Optimized quantification and a qualification transitions were selected for each lipid of interest and the respective deuterated internal standards. Calibrations curves, obtained in the same conditions, were used to convert into concentrations the ratio between the signal of the analyte over the signal of the internal standard.

#### Faecal analyses

Stools were collected from a Commode Specimen Container (Covidien, Minneapolis, MI, US) placed over the toilet seat opening. A nut‐sized section of faecal material was transferred by the patient to Sarstedt Feces Tube 76 × 20 mm (Sarstedt, Nürmbrecht, Germany), immediately stored in the patient's fridge at 2–8°C, and transported to the laboratory within 24 h. Two aliquots of 80–120 mg, made into 2 mL safe‐lock (Eppendorf, Hamburg, Germany) tubes (for later faecal IgA measurements), and the remaining faecal material in the original Feces Tube (for later DNA extraction) were kept frozen at −80°C until processing.

The aliquots were used to measure faecal IgA levels by enzyme‐linked immunosorbent assay according to the instruction of the manufacturer, in duplicates (IBL International, Hamburg, Germany). All measurements were performed simultaneously at the end of the study.

The faecal material in the Feces Tubes was used for the metataxonomic analysis of faecal microbiota. DNA was extracted from 120–200 mg stools using ZymoBIOMICS DNA Miniprep Kit (Zymo Research, Irvine, CA, USA). Negative extraction controls were performed using the same kit but omitting the addition of stools. Purified DNA was quantified using the Qubit dsDNA BR Assay Kit (Thermo Fisher Scientific, Waltham, MA, USA) and stored at −20°C.

The V3–V4 region of the bacterial 16S rRNA genes was amplified using 3 ng of extracted DNA in a 25 μL volume of ZymoTaq PreMix (ZymoBIOMICS) containing each 0.4 μM 341F 5′‐CCTACGGGNGGCWGCAG‐3′ and 805R 5′‐GACTACHVGGGTATCTAAKCC‐3′ primers. The PCRs were carried out with an initial denaturation at 95°C for 3 min, followed by 29 cycles of denaturation at 95°C for 30 s, annealing at 51°C for 30 s, and extension at 72°C for 60 s, and a final extension at 72°C for 7 min. Duplicate PCRs of each sample were combined and run on a 2100 Bioanalyzer (Agilent Technologies, Santa Clara, CA, USA) for quality analysis and quantification. The amplicon barcoding/purification, the construction, and sequencing library were performed at LGC Genomics (Berlin, Germany) as described previously.^15^

Primer and adapter trimmed sequencing reads were quality filtered and joined using PEAR v.0.9.11 (‐m 470 ‐n 390 ‐t 150 ‐v 10 ‐q 33 ‐p 0.0001 ‐u 0).^17^ Merged sequence reads were clustered into zero‐radius operational taxonomic units (zOTUs) using UNOISE3 from USEARCH v.10.0.240,^18^ which resolves differences of as little as one nucleotide and removes out putative chimeric sequences.^19^ zOTUs were classified using EzBioCloud 16S rRNA gene sequence database (as of 19 August 2019)^17^ via MOTHUR's command classify.seqs (method = wang, cut‐off = 80). From the sample dataset, we removed zOTUs matching any of the following criteria: (i) remained unclassified at the bacterial phylum level, (ii) presented <90% identity to the reference EzBioCloud database sequences as revealed by USEARCH^18^ (‐usearch_global ‐id 0.90 ‐query_cov 0.99), (iii) were represented by ≤10 counts, or (iv) had >100× higher average relative abundance in negative controls than in samples (four zOTUs).

### Statistics

#### Non‐metataxonomic data

Statistics were performed with Stata Release 16.1. The normality of distribution of serum cytokines, LPS, and faecal IgA was checked by Shapiro–Francia tests on each time point. As they were not normally distributed, results are shown as median (percentile 25 and 75). Comparisons between groups at baseline were performed with Wilcoxon–Mann–Whitney rank‐sum tests. Significance was set at *P* < 0.05 and adjusted for multiple testing by the Benjamini–Hochberg method.^20^

The impact of glycine and BCAA on serum levels of cytokines, surrogates of gut permeability, and of appetite mediators and on faecal IgA levels was evaluated by multiple mixed linear regression models.^21^ For this purpose, each parameter was normalized using the ‘ladder’ command on Stata. The regressions were performed following the latest CONSORT statement for randomized crossover trials in a per‐protocol analysis and did not take into account a carry‐over effect. The models included age, sex, supplementation (BCAA vs. glycine), month of supplementation (0 and 4 in each period), and period (Period 1: first 4 months; Period 2: last 4 months), as well as subjects as random intercepts. Significance, set at *P* < 0.05, was again corrected for multiple testing by the Benjamini–Hochberg method.^20^

In the significant models that found a statistical difference between both types of supplementations, we added a ‘supplementation × months’ interaction term to evaluate whether time played a role. In these models, we also performed the multiple mixed linear regression models separately by type of supplementation, in order to evaluate the impact of glycine or BCAA on the evolution of a given parameter, as compared with baseline.

#### Metataxonomic data

Illumina sequencing of 108 stool samples from 27 patients and 3 no‐sample controls generated 9 318 530 primer‐clipped read pairs. The filtered dataset contained 9 066 637 merged reads, with a median of 80 927 (41 940–207 850) per sample. Sequencing data were submitted to the European Nucleotide Archive (www.ebi.ac.uk/ena; study number: PRJEB43505).

Overall intra‐individual and inter‐individual similarities between bacterial communities were assessed in PRIMER v.7.0.13 (PRIMER‐e, Plymouth, UK) using Bray–Curtis similarity matrix, based on the square‐root‐transformed relative abundances of zOTUs. To assess differences in overall microbiota taxonomic composition, we used principal coordinate analysis and permutational multivariate analysis of variance (PERMANOVA).

Shannon diversity index (*H*′ = −Σ*pi* × ln*pi*), evaluating species diversity, was calculated from the relative abundance of zOTUs (*pi*) after rarefying the dataset to the same sequencing depth (41 500) across all samples using the ‘rrarefy’ command of the R vegan package. For calculating zOTU richness, a zOTU was considered present in a sample if represented by at least one read.

To compare the relative abundance of individual taxa (from phylum down to the zOTU level) between the start and the end of each supplementation, we used the analysis of composition of microbiomes v.2.1^22^ with the following settings: adjusted = F, repeated = F, paired.test_ancom = paired, multcorr = 2 (‘less stringent’ multiple comparison correction), sig = 0.05, and prev.cut = 0.75 (features not observed in ≥ 75% samples were omitted). The results that passed the 0.6 threshold when analysing both groups together were considered significant. Wilcoxon signed‐rank test was used to assess the significance of intra‐individual differences in bacterial diversity between sampling points.

#### Sample size calculation

Power calculations were performed for the primary outcome (gut microbiota composition) and secondary outcomes, when data for power analysis were available in the literature. We reached the highest *N* when performing the power calculation for gut microbiota composition and therefore detail this calculation thereafter.

Calculation of the sample size for the primary outcome was based on the % differences of total operational taxonomic units published by Vaziri *et al*.,^23^ which was 13% higher in patients with end‐stage renal disease than in controls. We relied on this article because no longitudinal data on gut microbiota composition were available on haemodialysis patients. We had hypothesized that the gut microbiota of our patients under BCAA would normalize and be comparable with the controls of the study of Vaziri *et al*. and that the patients under placebo (glycine) would be comparable with patients with end‐stage renal disease. A sample size of 26 achieves 100% power to detect a difference of 0.13 using a two‐sided binomial test. Significance level was set at *P* < 0.05 with a power of 90%.

We estimated a dropout rate of 30% (10% for non‐compliance, 5% for kidney transplantation, and 15% for death). The final number of subjects needed in the study was calculated using the following published formula^20^: *N* final = *N* power∕((1—death risk)^*i*^ * (1—non‐compliance)^*i*^ * (1—transplantation)^*i*^), where *i* is equal to duration of the study, rounded up to 1 year in this study. Thus, in order to obtain the data of 26 patients completing the study *lege artis*, we needed to include 36 subjects.

## Results

**Figure 1:**
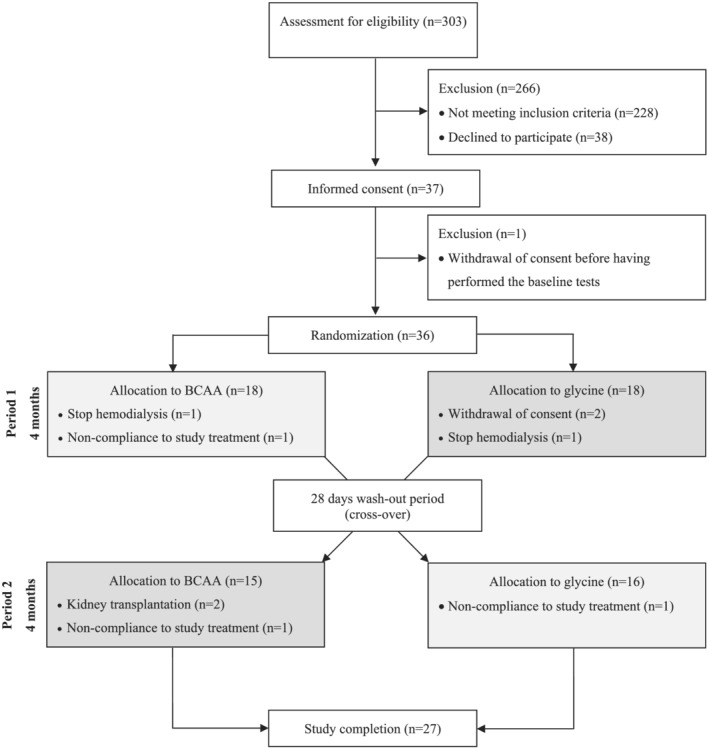
Consort flow diagram. BCAA, branched‐chain amino acid.

Screening was performed in 303 patients. Thirty‐seven patients were randomized, but one of them did not complete the baseline tests nor started the supplementation, and therefore, his supplementation was given to another patient. Nine patients did not complete the study for the reasons mentioned in *Figure* 1.

Twenty‐seven patients (11 women and 16 men) completed the study, which corresponds to 12 patients in the glycine–BCAA group (7 women and 5 men) and to 15 patients in the BCAA–glycine group (4 women and 11 men) (*Figure* 1). The aetiologies of kidney failure, described in our other article,^11^ were mostly hypertension (*n* = 17), diabetes (*n* = 9), chronic glomerulonephritis (*n* = 4), and polycystic kidney disease (*n* = 4), and some patients had a multifactorial aetiology. The patients had a mean age of 61.2 ± 13.7 years and a body mass index of 27.7 ± 5.1 kg/m^2^.

### Non‐metataxomic results

At baseline, there were no differences in surrogate serum markers of systemic inflammation, intestinal permeability, as well as in appetite mediators, and endocannabinoids levels between both groups (*Table* 1). The differences of these parameters between Months 4 and 0 of each supplementation, independently whether they started or ended with BCAA or glycine, are shown in Supporting Information, *Table* S2.

**Table 1:** Baseline plasma markers of inflammation, intestinal permeability, and of appetite mediators and endocannabinoids

	All patients (*n* = 27)		BCAA–glycine group (*n* = 15)^a^		Glycine–BCAA group (*n* = 12)^a^		
	Median	P25–75	Median	P25–P75	Median	P25–P75	*P* ^b^
Systemic inflammation							
Serum C‐reactive protein (g/L)^c^	4.0	(2.5–12.8)	4.2	(3.1–24.6)	2.6	(2.3–6.3)	0.091
Serum interleukin‐6 (pg/mL)	1.3	(0.7–3.1)	1.4	(0.7–3.6)	1.2	(0.7–1.7)	0.495
Serum interleukin‐10 (pg/mL)	0.1	(0.1–0.3)	0.1	(0.1–0.3)	0.1	(0.1–0.2)	0.733
Serum tumour necrosis factor‐α (pg/mL)	2.8	(2.1–3.9)	2.8	(2.4–3.5)	2.7	(1.8–3.9)	0.733
Faecal IgA (μg/mL)	1600	(325.0–3700.0)	2563.0	(425.0–6775.0)	1062.5	(287.5–3144.0)	0.354
Intestinal permeability							
Serum lipopolysaccharides (ng/mL)	76.6	(62.0–112.9)	76.6	(49.6–110.8)	76.9	(66.4–112.9)	0.626
Serum glucagon‐like peptide 2 (ng/mL)	8.8	(6.0–10.1)	8.8	(6.0–10.2)	8.2	(5.9–9.8)	0.733
Appetite mediators							
Total ghrelin (pg/mL)	460.3	(293.7–1102.6)	619.7	(291.3–1798.4)	437.7	(298.1–879.3)	0.495
Active ghrelin (fmol/mL)	12.8	(7.5–24.0)	12.8	(7.3–24.0)	13.3	(8.0–24.3)	0.770
Leptin (pg/mL)	33 507.5	(7004.2–70 894.6)	12 776.4	(6418.8–70 894.6)	40 589.1	(21 237.8–67 596.5)	0.262
Active glucagon‐like peptide 1 (pM)	0.3	(0.2–0.6)	0.4	(0.2–0.6)	0.2	(0.1–0.4)	0.143
Cholecystokinin (pg/mL)	353	(262.5–444.9)	310.0	(262.5–420.5)	407.3	(264.4–476.4)	0.329
Neuropeptide Y (pg/mL)	77.0	(54.8–108.8)	71.0	(65.6–108.8)	83.1	(49.5–109.1)	0.733
Peptide YY (pg/mL)	175.4	(114.8–256.4)	179.2	(114.8–310.8)	156.3	(107.2–225.2)	0.435
Endocannabinoids							
Arachidonoylglycerol (1‐AG and 2‐AG) (ng/mL)	3.7	(3.5–4.0)	3.8	(3.6–4.0)	3.7	(3.4–4.2)	0.591
Oleoylglycerol (1‐OG and 2‐OG) (ng/mL)	61.0	(30.4–84.6)	69	(52.4–88.3)	53.1	(24.1–63.8)	0.143
Anandamide (ng/mL)	2.2	(1.7–3.0)	2.2	(1.7–3.0)	2.4	(1.8–2.9)	0.961
*N*‐Oleoylethanolamine (ng/mL)	2.0	(1.4–2.4)	2.0	(1.4–2.7)	1.9	(1.5–2.2)	0.807
*N*‐Palmitoyethanolamine (ng/mL)	4.7	(3.7–5.1)	4.7	(3.5–5.1)	4.7	(4.1–5.3)	0.807
*N*‐Linoleoylethanolamine (ng/mL)	1.2	(0.8–1.4)	1.2	(0.5–1.4)	1.2	(1.1–1.8)	0.329
*N*‐Stearoylethanolamine (ng/mL)	0.6	(0.5–0.9)	0.7	(0.6–0.8)	0.6	(0.5–0.9)	0.626

BCAA, branched‐chain amino acid; P, percentile.

^a^ The BCAA–glycine group started with the BCAA supplementation, and the glycine–BCCA group started with the glycine supplementation.

^b^ Wilcoxon rank‐sum test.

^c^ *n* = 26 (one person missing in the glycine–BCAA group).

Multiple mixed linear regression models were significant only for IL‐6, GLP‐1, CCK, and PYY (*Table* S2 and stayed significant after correction of the *P*‐value for multiple testing. Although BCAA increased significantly GLP‐1 and PYY, there was no significant impact of the ‘month of supplementation’ (*Table* S2) and of the interactions ‘supplementation × months’ (*P* = 0.710 and *P* = 0.985, respectively). When performing the multiple mixed linear regression models separately by type of supplementation, there was also no impact of the ‘month of supplementation’ (0 or 4) on GLP‐1 (*P* = 0.098 under BCAA; *P* = 0.257 under glycine) and PYY (*P* = 0.773 under BCAA; *P* = 0.728 under glycine), confirming that time did not play a role in these differences. The result of the multiple linear regression models for CRP was shown in another article.^11^ The plasma levels of endocannabinoids were not affected by the type of supplementation.

### Metataxonomic results

#### Overall intra‐individual and inter‐individual microbiota similarity

**Figure 2:**
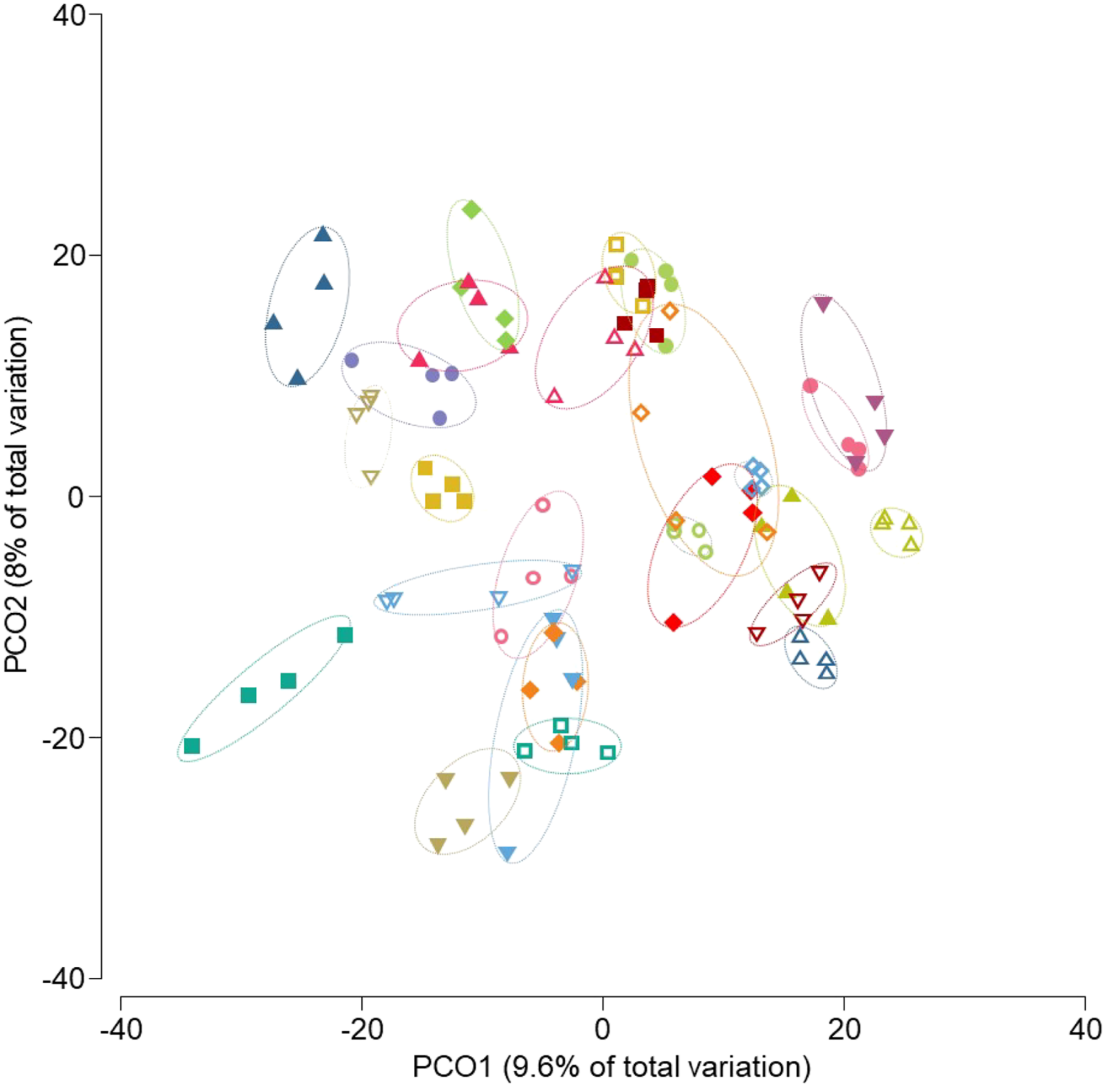
Principal coordinate analysis of Bray–Curtis similarity of bacterial communities showing that microbiota strongly cluster by subject. The analysis was based on the square‐root‐transformed relative abundance of zero‐radius operational taxonomic units. Four faecal samples were collected for each patient, at Months 0 and 4 of the glycine supplementation and Months 0 and 4 of the branched‐chain amino acid (BCAA) supplementation. As the overall microbiota similarity did not differ significantly between time points and supplementation, we omitted this legend on the figure for more clarity. The open symbols correspond to the samples of patients in the glycine–BCAA group and the filled symbols to the samples of patients in the BCAA–glycine group.

Principal coordinate analysis of Bray–Curtis similarity showed that microbiota strongly cluster by subject (*Figure* 2). PERMANOVA analysis confirmed statistically significant differences between individuals (global test *P* = 0.0001; pairwise tests *P* = 0.0245–0.0333). In contrast, no significant overall microbiota changes were found between different time points in each of the groups. Similarly, when the two groups were considered together, the PERMANOVA test did not reveal significant changes in the microbiota under glycine or BCAA supplementation. The intra‐individual Bray–Curtis similarity between the microbiota collected at the beginning and at the end of supplementation was not significantly different under glycine vs. BCAA supplementation when the two groups (glycine–BCAA and BCAA–glycine) were considered individually (Wilcoxon signed‐rank test, *P* = 0.804 and *P* = 0.3804, respectively) or together (*P* = 0.7317).

#### Microbiota diversity

**Figure 3:**
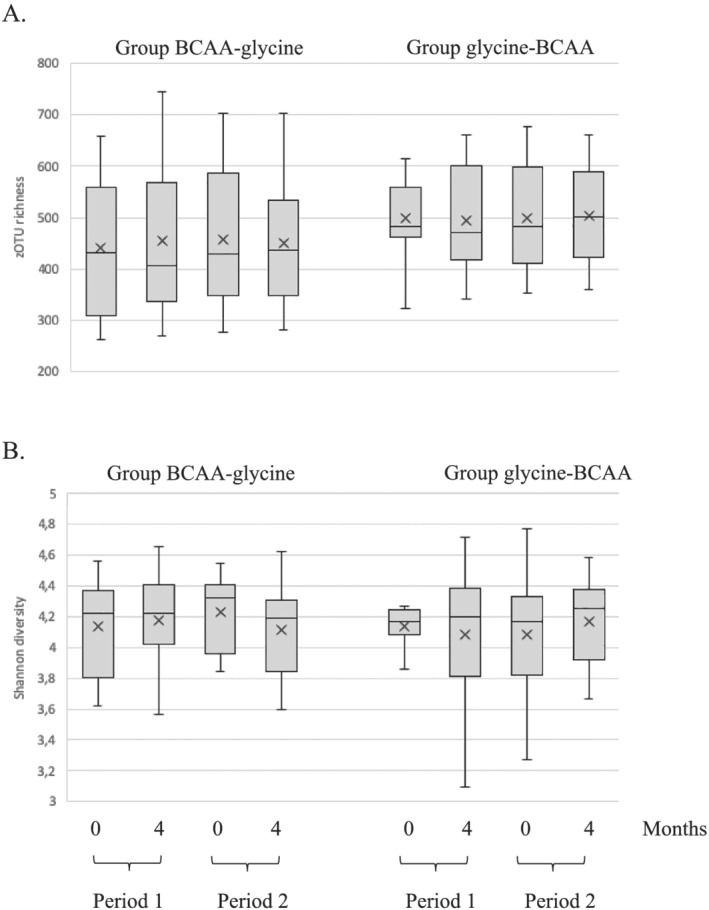
Alpha diversity of faecal bacterial communities. The dataset was rarefied to 41 500 reads prior to calculating *(A)* zero‐radius operational taxonomic unit (zOTU) richness and *(B)* Shannon diversity index; 0 and 4 indicate the sampling month. BCAA, branched‐chain amino acid.

Bacterial alpha diversity measured as Shannon diversity index and zOTU richness remained stable over the 9 month period in both groups (*Figure* 3). The only significant change observed was a decrease in the median value of the Shannon diversity index in the BCAA–glycine group when the samples collected at the end of glycine supplementation were compared with those collected at the start of the glycine supplementation (*P* = 0.0302). However, this decrease during the glycine treatment was rather modest (4.322; Q1–Q3: 3.985–4.397 vs. 4.189; Q1–3: 3.8785–4.279) and was no longer significant after combining data from both groups.

#### Taxa differences between groups

**Figure 4:**
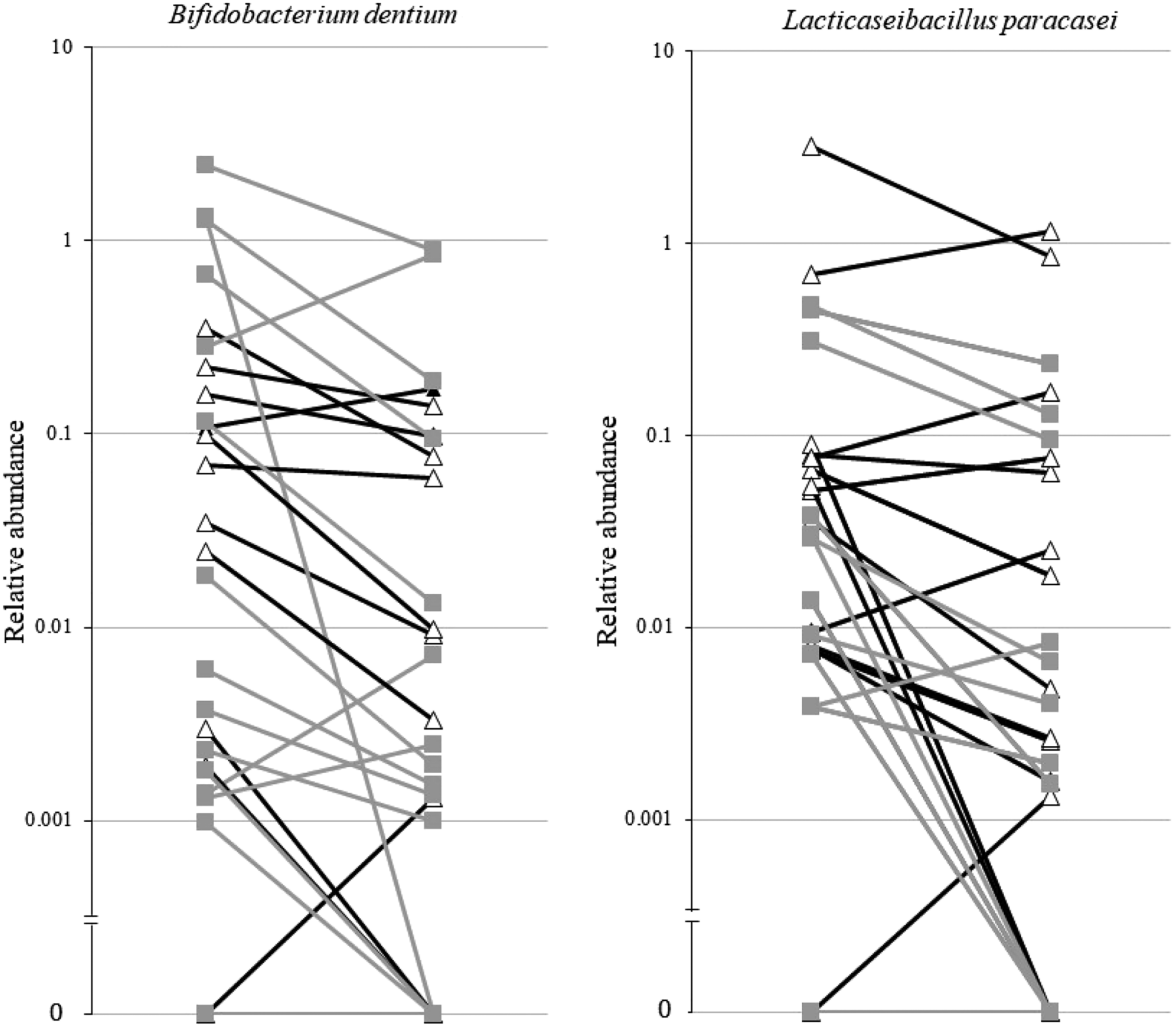
Line plots showing the decrease of the relative abundance of *Bifidobacterium dentium* and *Lacticaseibacillus paracasei*, between Months 0 and 4, under branched‐chain amino acid (BCAA). Black lines represent the group treated first with BCAA in the first 4 months of the study and the grey lines the group treated with BCAA in the second 4 months of the study.

The abundance of *Bifidobacterium dentium* significantly decreased between Months 0 and 4 of the BCAA supplementation (0.0247; Q1–Q3 0.002–0.191 vs. 0.003; Q1–Q3 0.001–0.086), as did the abundance of *Lacticaseibacillus paracasei* (0.030; Q1–Q3 0.008–0.078 vs. 0.004; Q1–Q3 0.001–0.070) (*Figure* 4). When considering the glycine–BCAA group separately, BCAA supplementation still led to a significant decrease in the abundance of *L. paracasei* (0.021; Q1–Q3 0.006–0.105 vs. 0.003; Q1–Q3 0–0.030) but also to an increase of *Ruminococcus g_2* (0.916; Q1–Q3 0.386–2.189 vs. 2.857; Q1–Q3 2.068–3.816). In the same group, *Akkermansia muciniphila* abundance decreased between the beginning and the end of glycine supplementation (0.039; Q1–Q3 0.002–1.642 vs. 0.001; Q1–Q3 0–0.061). No significant changes occurred in the BCAA–glycine group.

## Discussion

This randomized, placebo‐controlled, double‐blind crossover study showed that serum levels of cytokines, intestinal permeability markers, appetite mediators, endocannabinoids, and faecal levels of IgA did not change between the oral supplementations of glycine and BCAA, and between the start and end of each supplementation considered separately. Overall faecal microbiota composition and microbial diversity did not change with the glycine or BCAA supplementation. However, the abundance of *L. paracasei* and *B*. *dentium* decreased with the BCAA supplementation. As reported elsewhere,^11^ calorie and protein intakes did not differ between the groups at baseline, whether starting with glycine or BCAA supplementation. They did not differ either between groups during the follow‐up, whatever the supplementation was, and they remained between 21–23 kcal/kg/day and 0.80–0.82 g/kg, respectively, throughout the study.

### Amino acids and gut microbiota

It has been suggested that nutrition could affect the composition of the gut microbiota and its function in patients with chronic kidney disease.^24^ Although several studies have shown a beneficial impact of an oral amino acid supplementation on the nutritional status of these patients,^3^ no human study has evaluated its impact on the faecal microbiota. *In vitro*, amino acids can be metabolized by intestinal bacteria.^25^ In mice, BCAA increased the faecal abundance of *A*. *muciniphila* and *Bifidobacterium*, decreased that of *Enterobacteriaceae*, and reduced the serum levels of lipopolysaccharide‐binding protein, which is known to bind LPS and likely reflects inflammatory state.^26^ So far, there are also no studies describing changes in the composition of the gut microbiota in response to glycine intake. Here, we show that the glycine supplementation had no impact on the gut microbiota, while the BCAA supplementation decreased the abundance of two beneficial bacteria: *L. paracasei*, a microorganism traditionally used as probiotic, and *B*. *dentium*, a species that enhances the intestinal mucus layer and goblet cell functions.^27^ At first sight, this suggests that there is no role for the gut microbiota in the increase of fat‐free mass after glycine administration. Alternatively, it is possible that glycine supplementation prevents the loss of *L. paracasei and B*. *dentium* in haemodialysis patients and that the decrease of these bacteria with BCAA is involved in the loss of fat‐free mass, but this needs further research.

Several clinical trials have focused on the impact of known prebiotics, defined as a ‘substrate that is selectively utilized by host microorganisms conferring a health benefit’, or synbiotics, in dialyzed patients. Prebiotics in the form of non‐digestible carbohydrates were shown to decrease the abundance of faecal *Bacteroides thetaiotaomicron* without affecting overall microbial community, and faecal bacterial metabolites associated with cardiovascular diseases as indoxyl sulfate and *p*‐cresyl sulfate.^28^ Synbiotics increased the bifidobacteria count and the levels of faecal butyric acid but decreased serum indoxyl sulfate and *p*‐cresyl sulfate.^29^ Although our study enrolled the same type of patients and was comparable in terms of included number of patients, the supplementation lasted longer and was different from the aforementioned studies, thereby likely explaining the differences in gut microbiota changes.

### Amino acids, cytokines, gut permeability, and appetite mediators

The absence of impact of BCAA and glycine on serum levels of cytokines, surrogate markers of gut permeability, appetite mediators, and of faecal levels of IgA was surprising.

Inflammation is involved in muscle wasting. As we had found an increase in fat‐free mass with glycine but not with BCAA,^11^ we expected a decrease in the serum levels of pro‐inflammatory cytokines. This would have been in line with trials involving patients with chronic diseases other than haemodialysis, which showed a decrease of serum TNF‐α and IL‐6 with glycine supplementation.^30^, ^31^ The fact that we could not find a decrease may be related to the disease of our patients. Indeed, these molecules are retained in end‐stage renal disease and haemodialysis has a limited capacity to clear them from the circulation.^32^ Faecal IgA, secreted by the intestinal mucosa, could reflect the immunological response to pathogens and be a marker of intestinal inflammation,^33^ although the significance of its stool concentrations has not yet been well established.

There are hints in the literature that both types of supplementation could potentially decrease the intestinal epithelial permeability. Indeed, orally administered BCAAs are used in the splanchnic tissues.^34^ They also stimulate the synthesis and release of glutamine by the skeletal muscle *in vitro*,^35^ which is the preferred substrate of rapidly dividing cells as enterocytes.^36^ Regarding glycine, it has been shown that it reduces the oxidative stress on intestinal epithelial cells.^37^ Thus, either BCAA and glycine truly do not influence gut permeability in HD patients or the surrogate makers of gut permeability used in our study, that is, LPS and GLP‐2, lack sensitivity to detect small changes.

It has been shown previously that BCAA improves appetite in HD patients^11^ and in patients with liver cirrhosis^37^ or cancer.^38^ The mechanisms have not yet been untangled. No literature is available on the impact of glycine on appetite. We had hypothesized that anabolism is promoted through a dysbalance of appetite mediators in favour of orexigenic mediators^20^ and through an increase in serum endocannabinoids.^39^ We could not show any impact of BCAA or glycine on the serum levels of these mediators, but it is important to note that we could not find an increase in appetite and nutritional intake either^11^ in contrast to the aforementioned studies.

### Strengths and limitations

The strength of our study was the crossover design. This design is especially important knowing the higher inter‐individual variability of gut microbiota than the intra‐individual variability over time, and it allowed each subject to be evaluated for both treatments. The dropout rate for non‐compliance to study supplementation was low (*n* = 3). We had simultaneous measurements and follow‐up of several surrogate parameters of the gut barrier including gut microbiota, in addition to clinical outcomes, which were described in a previous paper.^11^

One limitation of the study was that we had no control group, as the glycine supplementation was originally foreseen as placebo. We could not perform analysis separately for women and men due to the small sample size. We did not measure microbial metabolites. We have considered the possibility of confounding factors for gut microbiota differences. Bifari *et al*. have reported that the impact of amino acid supplementation on gut microbiota depends on lifestyle factors as diet and physical activity, as well as on health and disease state.^40^ However, in our study, we had advised our patients to pursue their habitual dietary regimen and physical activity during the study, and we have shown that their calorie and protein intake, and their physical activity, assessed by 7 day pedometry, did not vary significantly over time.^11^ Although we cannot exclude that changes in health state and medication may have occurred, our patients were under chronic HD and experienced only few adverse events during the study time.^11^

## Conclusion

This study showed that the BCAA and glycine supplementation did not affect the serum levels of cytokines, appetite mediators, intestinal permeability markers, and faecal IgA. Overall, the faecal microbiota composition and microbial diversity did not change with the glycine or BCAA supplementation, but the abundance of *L. paracasei* and *B*. *dentium* decreased with the BCAA supplementation.

## Funding

This study was financially supported by the Swiss National Science Foundation (Schweizerischer Nationalfonds zur Förderung der Wissenschaftlichen Forschung; 320030_163144), Alfred and Alice Lachmann Foundation, and Fresenius Kabi Deutschland GmbH. The funding source had no role in the design and conduct of the study, analysis or interpretation of the data, or preparation of final approval of the manuscript prior to publication. P.D.C. is senior research associate from the FRS‐FNRS (Fonds De La Recherche Scientifique ‐ FNRS, Belgium) and received grants from the FRS‐FNRS (Projet de Recherche PDR‐convention: FNRS T.0030.21‐P; FRFS‐WELBIO: WELBIO‐CR‐2019C‐02R).

## Conflict of interest

L.G. has received grants from the Swiss National Foundation, Alfred and Alice Lachmann Foundation, and Fresenius Kabi; speaker honoraria from Fresenius Kabi; advisory honoraria from Baxter and Abbott; and travel grants from Nestlé Health Science and Abbott. P.D.C. is a co‐founder of A‐Mansia Biotech S.A. (Belgium) and owner of several patents concerning the use of specific bacteria or components on the treatment of obesity, diabetes, and cardiometabolic disorders. D.T. has received grants from Fresenius Medical Care, Amgen, and Baxter; speaker honoraria from Fresenius Medical Care, B Braun, Abbott International, Baxter, Genzyme, and Sanofi Aventis; travel grants for Fresenius Medical Care, Amgen, and Vifor; and a grant for teaching material from Abbott International. M.P., C.S., N.M., J.M., I.B., A.W.‐G., V.L., L.G., F.R.H., and J.S. have no conflicts of interest.

## Supporting information


**Table S1.** Impact of 4 months of supplementation with BCAA or glycine on plasma markers of inflammation, intestinal permeability and of appetite mediators and endocannabinoids (results shown as differences between month 4 and month 0 of each supplementation.
**Table S2.** Multiple mixed linear regressions including period, supplementation, months, age and sex as fixed effects, and subjects as random intercepts, to predict systemic inflammation and intestinal permeability. Only the significant models are shown.
