# Supplementary material for: Gut barrier and microbiota changes with glycine and branched‐chain amino acid supplementation in chronic haemodialysis patients
Source: J Cachexia Sarcopenia Muscle. 2021 Sep 18;12(6):1527–39. doi: 10.1002/jcsm.12781 (PMC8718035; doi:10.1002/jcsm.12781)
Supplement: Supplementary file 1 — Table S1. Impact of 4 months of supplementation with BCAA or glycine on plasma markers of inflammation, intestinal permeability and of appetite mediators and endocannabinoids (results shown as differences between month 4 and month 0 of each supplementation. Table S2. Multiple mixed linear regressions including period, supplementation, months, age and sex as fixed effects, and subjects as random intercepts, to predict systemic inflammation and intestinal permeability. Only the significant models are shown. [file JCSM-12-1527-s001.docx]

**Supplemental table 1:** Impact of 4 months of supplementation with BCAA or glycine on plasma markers of inflammation, intestinal permeability and of appetite mediators and endocannabinoids (results shown as differences between month 4 and month 0 of each supplementation.

|  | BCAA | | Glycine | |
| --- | --- | --- | --- | --- |
|  | Month 4 - Month 0 | | Month 4 - Month 0 | |
|  | median | (P25; P75) | median | (P25; P75) |
| **Systemic inflammation** |  |  |  |  |
| Serum C-reactive protein (g/l) | -1.00 | (-4.5; 0.00) | -0.40 | (-1.40; 3.60) |
| Serum interleukin-6 (pg/ml) | -0.18 | (-1.39; 0.24) | 0.03 | (-0.4; 0.25) |
| Serum interleukin-10 (pg/ml) | -0.01 | (-0.01; 0.03) | -0.01 | (-0.04; 0.02) |
| Serum tumor necrosis factor-α (pg/ml) | -0.11 | (-0.49; 0.33) | -0.09 | (-0.43; 0.49) |
| Fecal IgA (µg/ml) | -53.50 | (-1753.80; 200.00) | -69.00 | (-783.00; 1088.00) |
| **Intestinal permeability** |  |  |  |  |
| Serum lipopolysaccharides (ng/ml) | -1.76 | (-15.81; 3.00) | -2.10 | (-11.09; 7.03) |
| Serum glucagon-like peptide 2 (ng/ml) | 0.58 | (-0.59; 1.69) | -0.78 | (-1.35; 0.49) |
| **Appetite mediators** |  |  |  |  |
| Total ghrelin (pg/ml) | -10.39 | (-70.44; 172.89) | 26.24 | (-123.91; 216.41) |
| Active ghrelin (fmol/ml) | -0.61 | (-5.85; 1.16) | 0.90 | (-2.42; 3.85) |
| Leptin (pg/ml) | 3606.52 | (-1468.99; 11468.05) | -751.12 | (-14604.35; 1512.02) |
| Active glucagon-like peptide 1 (pM) | -0.01 | (-0.23; 0.16) | -0.04 | (-0.22; 0.04) |
| Cholecystokinin (pg/ml) | 16.2 | (-26.72; 52.96) | -0.20 | (-56.59; 47.09) |
| Neuropeptide Y (pg/ml) | 1.44 | (-10.43; 8.27) | -4.25 | (-15.72; 11.78) |
| Peptide YY (pg/ml) | 15.90 | (-2252; 43.37) | 1.91 | (-36.54;50.41) |
| **Endocannabinoids** |  |  |  |  |
| Arachidonoylglycerol (1-AG & 2-AG)(ng/ml) | -0.01 | (-0.24; 0.24) | 0.07 | (-0.15; 0.17) |
| Oleoylglycerol (1-OG & 2-OG)(ng/ml) | -0.60 | (-26.77; 16.11) | 3.89 | (-20.3; 27.5) |
| Anandamide (ng/ml) | 0.30 | (-0.51; 0.73) | 0.13 | (-0.40; 0.53) |
| N-Oleoylethanolamine (ng/ml) | -0.01 | (-0.38; 0.67) | 0.08 | (-0.21; 0.28) |
| N-Palmitoyethanolamine (ng/ml) | 0.02 | (-1.14; 1.53) | -0.19 | (-1.14; 1.03) |
| N-Linoleoylethanolamine (ng/ml) | 0.05 | (-0.54; 0.47) | 0.01 | (-0.43; 0.59) |
| N-Stearoylethanolamine (ng/ml) | 0.03 | (-0.13; 0.21) | 0.11 | (-0.16; 0.17) |

**Supplemental table 2**: Multiple mixed linear regressions including period, supplementation, months, age and sex as fixed effects, and subjects as random intercepts, to predict systemic inflammation and intestinal permeability. Only the significant models are shown.

|  |  | Coefficient | (95% CI) | p | p model |
| --- | --- | --- | --- | --- | --- |
| **Interleukin-6 (pg/ml, log)** |  |  |  |  | 0.002 |
| Supplementation | Glycine |  |  |  |  |
|  | BCAA | 0.84 | (0.70,1.00) | 0.055 |  |
| Months | 0 |  |  |  |  |
|  | 4 | 0.87 | (0.73,1.05) | 0.146 |  |
| Period | 1 |  |  |  |  |
|  | 2 | 0.77 | (0.64,0.93) | 0.006 |  |
| Age |  | 1.02 | (1.00,1.04) | 0.014 |  |
| Sex | Women |  |  |  |  |
|  | Men | 0.69 | (0.42,1.14) | 0.145 |  |
| **Active glucagon-like peptide 1 (pM, log)** |  |  |  |  | 0.028 |
| Supplementation | Glycine |  |  |  |  |
|  | BCAA | 1.31 | (1.01,1.68) | 0.040 |  |
| Months | 0 |  |  |  |  |
|  | 4 | 0.78 | (0.61,1.00) | 0.054 |  |
| Period | 1 |  |  |  |  |
|  | 2 | 1.12 | (0.87,1.45) | 0.381 |  |
| Age |  | 1.03 | (1.00,1.05) | 0.039 |  |
| Sex | Women |  |  |  |  |
|  | Men | 0.77 | (0.38,1.54) | 0.457 |  |
| **Cholecystokinin (pg/ml, log)** |  |  |  |  | 0.021 |
| Supplementation | Glycine |  |  |  |  |
|  | BCAA | 1.02 | (0.94,1.09) | 0.686 |  |
| Months | 0 |  |  |  |  |
|  | 4 | 1.02 | (0.95,1.10) | 0.589 |  |
| Period | 1 |  |  |  |  |
|  | 2 | 1.05 | (0.98,1.13) | 0.177 |  |
| Age |  | 0.98 | (0.97,0.99) | 0.002 |  |
| Sex | Women |  |  |  |  |
|  | Men | 0.87 | (0.66,1.16) | 0.356 |  |
| **Peptide YY (pg/ml, log)** |  |  |  |  | 0.002 |
| Supplementation | Glycine |  |  |  |  |
|  | BCAA | 1.14 | (1.05,1.25) | 0.002 |  |
| Months | 0 |  |  |  |  |
|  | 4 | 1.02 | (0.94,1.11) | 0.589 |  |
| Period | 1 |  |  |  |  |
|  | 2 | 1.15 | (1.05,1.25) | 0.002 |  |
| Age |  | 0.99 | (0.98,1.01) | 0.451 |  |
| Sex | Women |  |  |  |  |
|  | Men | 0.83 | (0.57,1.21) | 0.337 |  |

Significance of p value corrected to p<0.003 with the Benjamini-Hochberg method.
